# Bioplastic (poly-3-hydroxybutyrate)-producing *Massilia endophytica* sp. nov., isolated from *Cannabis sativa* L. ‘Cheungsam’

**DOI:** 10.1038/s41598-023-44976-w

**Published:** 2023-10-18

**Authors:** Doeun Jeon, Lingmin Jiang, Ki-Hyun Kim, Yuxin Peng, Donghyun Cho, Rae-Dong Jeong, Cha Young Kim, Jae Cheol Jeong, Jiyoung Lee

**Affiliations:** 1 Korean Collection for Type Cultures (KCTC), Biological Resource Center, Korea Research Institute of Bioscience and Biotechnology, Jeongeup, 56212 Republic of Korea; 2 Department of Applied Biology, Chonnam National University, Gwangju, 61186 Republic of Korea; 3 Department of Biosystems and Bioengineering, KRIBB School of Biotechnology, University of Science and Technology (UST), Yuseong, Daejeon, 34113 Republic of Korea

**Keywords:** Microbiology, Environmental sciences

## Abstract

A rod-shaped, motile, Gram-negative bacterial strain named DM-R-R2A-13^T^ was isolated from the plant *Cannabis sativa* L. ‘Cheungsam’. The phylogenetic analysis of the 16S rRNA gene sequence revealed that strain DM-R-R2A-13^T^ belongs to the family *Oxalobacteraceae* and is closely related to members of the genus *Massilia*, with *Massilia flava* (97.58% sequence similarity) and *Massilia armeniaca* (97.37% sequence similarity) being the closest members. The digital DNA-DNA hybridization (dDDH) values between strain DM-R-R2A-13^T^ and *Massilia flava* CGMCC 1.10685^T^ and *Massilia armeniaca* ZMN-3^T^were 22.2% and 23.3%, while the average nucleotide identity (ANI) values were 78.85% and 79.63%, respectively. The DNA G+C content was measured to be 64.6 mol%. Moreover, the bacterium was found to contain polyhydroxyalkanoate (PHA) granules based on transmission electron microscopy, indicating its potential to produce bioplastic. Genome annotation revealed the presence of PHA synthase genes (*phaC*, *phaR*, *phaP,* and *phaZ*), and the biopolymer was identified as poly-3-hydroxybutyrate (PHB) based on nuclear magnetic resonance (NMR) and Fourier transform infrared spectroscopy (FTIR) analyses. Using maltose as a carbon source, the strain produced PHB of up to 58.06% of its dry cell weight. Based on the phenotypic, chemotaxonomic, and phylogenetic characteristics, it has been determined that DM-R-R2A-13^T^ represents a novel species belonging to the genus *Massilia*. As such, the name *Massilia endophytica* sp. nov. is proposed for this newly identified species. The type strain is DM-R-R2A-13^T^ (= KCTC 92072^T^ = GDMCC 1.2920^T^).

## Introduction

The genus Italian was initially described by La Scola, who identified the species *Massilia timonae* in a blood sample from an immunocompromised patient with cerebellar lesions^1^. Since then, *Massilia* species are environmental microorganisms frequently found with plants. They have been isolated from a variety of plant-related sources, including soil^2^, flowers^3^, seeds^4^, and the roots of many species of plant^5^. *Massilia* species are adapted to effective surface colonization, including seed coat, newly sprouting radicles, root systems, and even extend into the hyphae of fungus *Pythium*^6^. Kämpfer et al. suggested that all *Naxibacter* species be reclassified as *Massilia* because of their identical chemotaxonomic characteristics^7^. As of now, the genus *Massilia* comprises 61 species with valid and correct names. *Massilia* is a Gram-negative, rod-shaped aerobic bacteria that have relatively high DNA G+C content (63.3–66.3 mol%)^5,8–15^. The major fatty acids in this genus are summed feature 3 (C_16:1_ω7c and/or C_16:1_ω6c) and C_16:0,_ while the major respiratory quinone is Q-8, and the major polar lipids are phosphatidylethanolamine (PE), phosphatidylglycerol (PG), and diphosphatidylglycerol (DPG)^16^. *Massilia* species possess diverse potential functions, such as the production of violacein^17,18^, degradation of benzene, toluene, ethylbenzene, and xylene (BTEX)^13^, solubilization of phosphate^19^, production of dimethyl disulfide^20^, adaptation to cold environments^21^, degradation of cellulose^10^, antibacterial activity^5^, degradation of chloroacetamide herbicide^22^, and accumulation of poly-3-hydroxybutyrate (PHB)^23^.

*Cannabis sativa* (commonly known as hemp) is among the earliest plants used by humans for food and medicine and to obtain fibers^24^. Hemp is a valuable source of cellulose and wood fibers and contains a variety of phytochemicals. The outer and inner stem tissues of hemp, in particular, have garnered attention due to their potential for producing materials such as bioplastics and concrete, respectively^25^. As global interest in hemp research has grown, investigating endophytes in hemp has become increasingly important.

Plastic materials made from petrochemicals are currently a significant environmental issue as they are non-biodegradable^26^. However, PHB, a polyester synthesized by microbes that belongs to the polyhydroxyalkanoate (PHA) family, offers a promising alternative to these non-biodegradable petroleum-derived plastics^27^. PHB is synthesized by many genera of bacteria, such as *Cupriavidus necator* (formerly *Ralstonia eutropha*)*,* using intracellular carbon and is accumulated in the cytoplasm^28^. PHB is biodegradable and biocompatible, and can be produced from sustainable carbon sources, making it an ideal substitute for petroleum-derived plastics like polypropylene and polyethylene^29^. To investigate the role of endophytic bacteria of *Cannabis sativa* L., we obtained 205 endophytic bacterial strains from the leaves and roots of the plant. One of the strains, designated DM-R-R2A-13^T^, was isolated from the roots of *Cannabis sativa* and found to belong to the genus *Massilia*. The combined phenotypic and genotypic analyses indicated that DM-R-R2A-13^T^ represents a novel species of genus *Massilia*, for which the name *Massilia endophytica* sp. nov. is proposed.

## Results and discussion

### Phylogenetic analysis using the 16S rRNA gene

**Figure 1:**
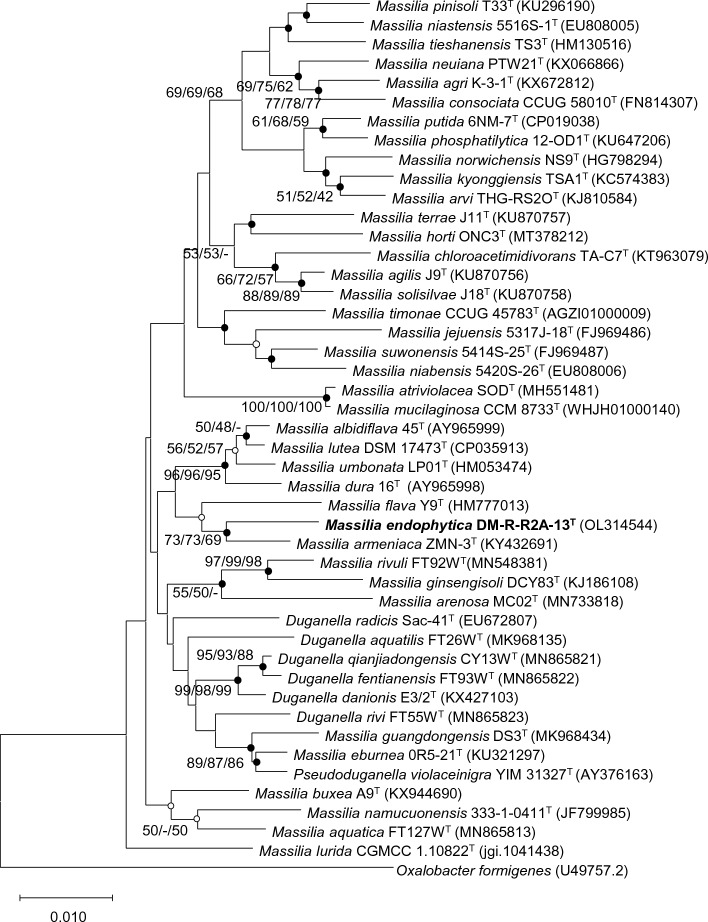
Phylogenetic tree of DM-R-R2A-13^T^ and other members of the genus *Massilia* using neighbor-joining phylogenetic tree based on 16S rRNA gene sequences. Solid circles at the nodes indicate generic branches where the relationships were also established by ML and ME algorithms, while open circles indicate branches recovered by either ML or ME algorithms. Scale bar represents 0.010 substitutions per position.

To investigate endophytes in hemp, we isolated 205 endophytic bacteria from the surface-sterilized leaves and roots of the plant. Phylogenetic analysis based on the 16S rRNA gene sequence revealed that strain DM-R-R2A-13^T^ belonged to the family *Oxalobacteraceae* and clustered with members of the genus *Massilia*. Strain DM-R-R2A-13^T^ showed its closest relationship with *M. flava* Y9^T^ (97.58% sequence similarity) and *M. armeniaca* ZMN-3^T^ (97.37%). Furthermore, the sequence similarities of strain DM-R-R2A-13^T^ with other members of the genus *Massilia* were less than 98%. In the phylogenetic trees, strain DM-R-R2A-13^T^ was well-clustered with *M. armeniaca* (Fig. 1). According to the minimum criteria for the classification of prokaryotes, the strain DM2-R-R1A-13^T^ can be considered a new species because its 16S rRNA gene sequence similarity is lower than 98.6%. The 16S rRNA gene sequence of strain DM-R-R2A-13^T^, which was 1469 bp long, was deposited in the GenBank database under the accession number OL314544.

### Genomic sequencing and annotation

To construct the genome of strain DM-R-R2A-13^T^, we used the SMRT Link de novo assembler, which produced a genome size of 5,387,550 bp and an N50 value of 12,697 bp. The genome comprises a single contig and had a coverage of 135.0 ×. (Fig. S1). The whole-genome sequence was deposited in GenBank (accession number: CP088952). The DNA G + C content of strain DM-R-R2A-13^T^ is 64.6%, which falls within the range of *Massilia* species. The annotation of the NCBI Prokaryotic Genome Annotation Pipeline (PGAP) identified a total of 4800 protein-coding genes and 94 RNA genes in the genome, including 6 5S rRNA genes, 6 16S rRNA genes, 6 23S rRNA genes, 72 tRNA genes, and 4 ncRNA genes. Cluster Orthologous Group (COG) annotation showed that a large proportion of the assigned COGs were classified as unknown (30.6% of total assigned COGs), while signal transduction mechanisms (7.2% of the total assigned COGs) and transcription (6.5% of the total assigned COGs) were among the most represented categories (Fig. S2).

**Figure 2:**
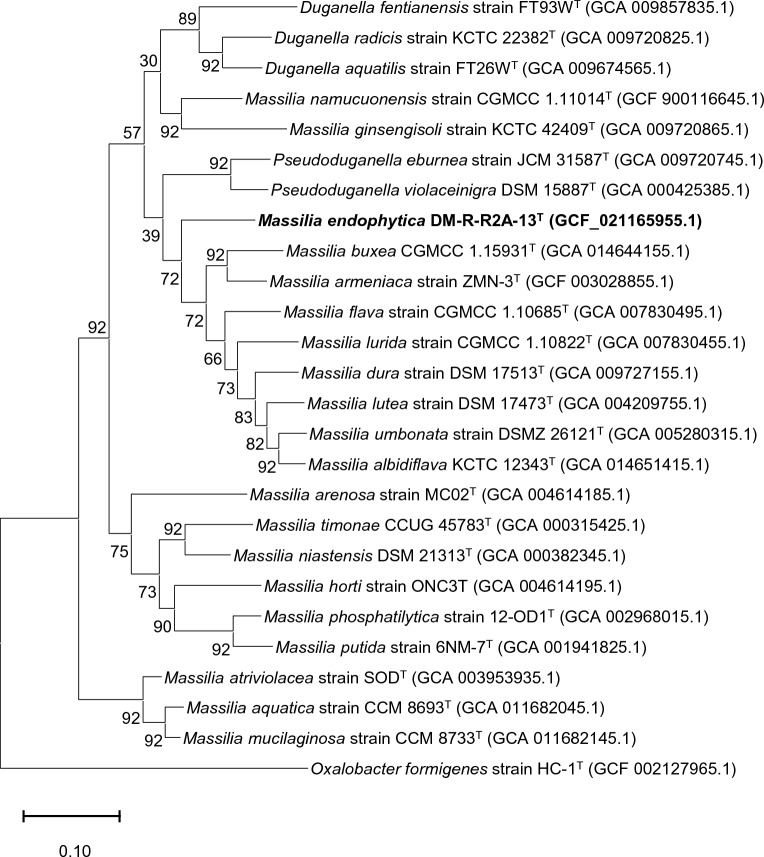
Phylogenomic tree of strain DM-R-R2A-13^T^ based on the multiple alignments of 92 bacterial core gene sequences as determined by UBCG. Scale bar represents 0.020 substitutions per position.

The dDDH values between the strain DM-R-R2A-13^T^ and the strains *Massilia flava* CGMCC 1.10685^T^ (VLKW01000000), *Massilia armeniaca* ZMN-3^T^ (CP028324), and *Massilia timonae* CCUG 45783^T^ (AGZI01000000) were 22.2%, 23.3% and 20.6%, respectively. The ANI values were 78.85%, 79.63%, and 75.83%, respectively. A whole-genome phylogenetic tree constructed by concatenated 92 core genes also supported the position of strain DM-R-R2A-13^T^ within the genus *Massilia* (Fig. 2).

### Phenotypic and chemotaxonomic characterization

**Figure 3:**
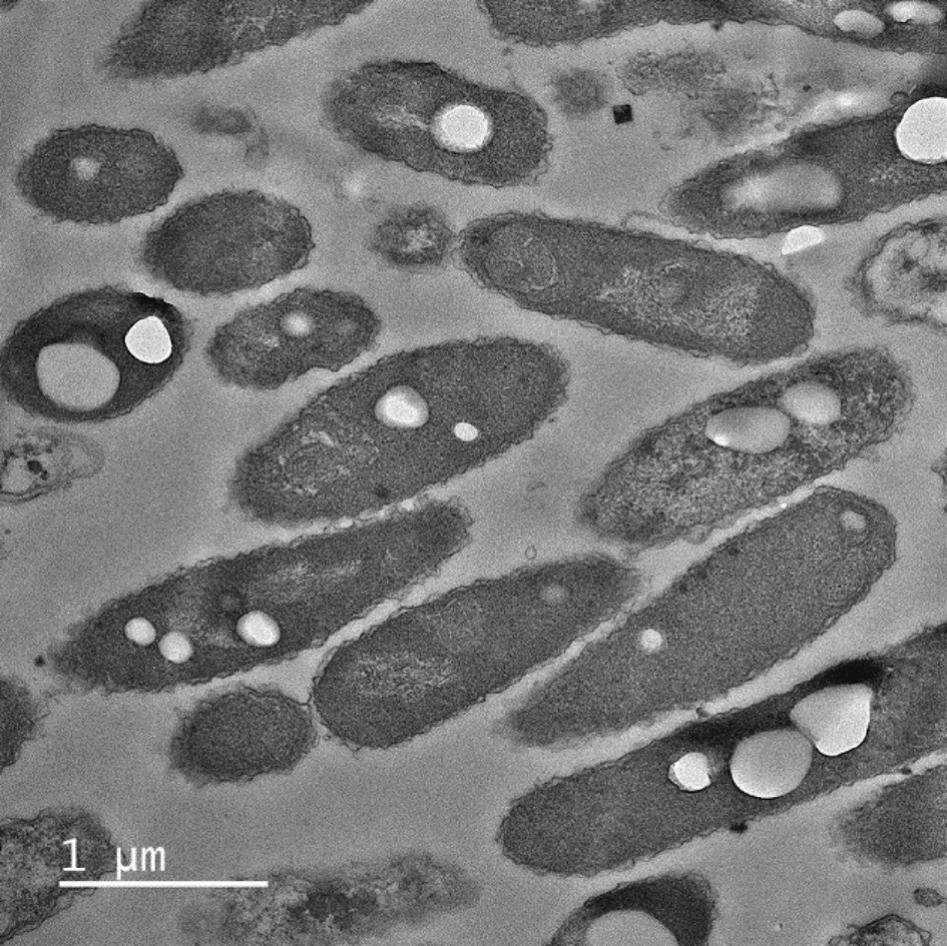
Transmission electron microscopy (TEM) image of strain DM-R-R2A-13^T^ containing polyhydroxybutyrate (PHB) granules.

Strain DM-R-R2A-13^T^, forming cream to light pink colonies, was a Gram-stain- negative, rod-shaped, and aerobic bacterium. It had motility by flagella (Fig. S3). The optimal conditions for cell growth were 30–37 °C and pH 6.0–7.5. It was noted that the strain grew well in the absence of sodium chloride (NaCl). Transmission-electron microscopy (TEM) analysis indicated several white particles such as PHB granules with high refraction (Fig. 3). Notably, the strain had phenotypic characteristics that helped distinguish it from closely related species, including the assimilation of *N*-acetyl-glucosamine. The strain was also distinguished from closely related species by its sensitivity to antibiotics such as kanamycin, tetracycline, and nalidixic acid (Table 1).

**Table 1:** Differential properties of strain DM-R-R2A-13^T^ and its closely related species of the genus *Massilia*.

Characteristics	1	2	3	4
Isolation source	*Cannabis sativa*	Soil^a^	Desert soil^b^	Blood of an immunocompromised patient^c^
Motility	Motile	ND	Motile^b^	Motile^c^
Colony color	Light pink	Yellow^a^	Apricot^b^	Straw^c^
Oxidase/catalase activity	+/+	+/+^a^	−/+^b^	+/+^c^
Enzyme activity (API ZYM)				
crystine arylamidase	+	+	−	+
Trypsin	−	−	−	−
α-chymotrypsin	w	−	−	w
α-galactosidase	−	+	+	−
β-galactosidase	+	+	−	−
α-glucosidase	+	+	+	−
β-glucosidase	+	−	−	+
Assimilation activity (API 20NE)				
D-glucose	−	−	−	−
L-arabinose	+	−	−	+
N-acetyl-D-galactosamine	+	−	−	−
D-maltose	+	−	−	+
Potassium gluconate	−	−	−	w
Malic acid	w	−	−	+
Trisodium citrate	−	−	−	+
Antibiotic sensitivity				
Kannamycin	+	+	+	+
Tetracyclin	+	+	+	+
Chloramphenicol	−	−	+	+
Nalidixic acid	+	−	+	+

Strains: 1, DM-R-R2A-13^T^; 2, *M. flava* KCTC 23535^T^; 3, *M. armeniaca* DSM 104676^T^; 4, *M. timonae* DSM 16850^T^;+, positive; –, negative; w, weakly positive; ND, no data available.

^a^Data from Wang et et al.^12^, ^b^Data from Ren et al.^10^; ^c^Data from La Scola et al.^1^.

The polar lipids in the strain were PE, PG and DPG (which are common in closely related species in the genus *Massilia*), one unidentified phospholipid (PL), and four unidentified aminolipids (AL) (Fig. S4). The dominant fatty acids observed in the strain were C_16:0_ and summed feature 3 (C_16:1_ω7c and/or C_16:1_ω6c) as shown in Table 2. Furthermore, the respiratory quinone analysis revealed the presence of ubiquinone Q-8, a characteristic quinone found in the genus *Massilia*. Based on these findings, it is proposed that strain DM-R-R2A-13^T^ be classified as a novel species of the genus *Massilia*, with the name *Massilia endophytica* sp. nov.

**Table 2:** Cellular fatty acid compositions (> 1%) of strain DM-R-R2A-13^T^ and its closely related species in the genus *Massilia*.

Fatty acid	1	2	3	4
C_10:0_	0.58	−	−	0.47
C_10:0_ 3-OH	4.76	2.41	4.92	4.46
C_12:0_	4.33	3.9	4.15	4.36
C_12:0_ 2-OH	2.8	−	−	2.02
C_12:0_ 3-OH	−	3.32	−	−
C_14:1_ω5c	0.6	0.74	1.34	0.67
C_14:0_	0.69	1.01	1.31	0.45
C_14:0_ 2-OH	−	2.54	3.06	−
iso-C_16:0_	0.32	−	−	−
C_16:0_	**33.71**	**30.94**	**27.47**	**34.24**
C_17:1_ω5c	0.62	1.02	−	−
C_17:0_ cyclo	4.61	3.57	−	0.77
summed feature 3*	**39.9**	**39.77**	**46.79**	**44.48**
summed feature 8*	7.09	**10.77**	**10.96**	8.08

Strains: 1, DM-R-R2A-13^T^; 2, *M. flava* KCTC 23535^T^; 3, *M. armeniaca* DSM 104676^T^; 4, *M. timonae* DSM 16850^T^; –, not detected. Major components (> 10%) are shown in bold. All data were obtained from the present study.

*Summed features are groups of one or two fatty acids that cannot be separated by GLC with the MIDI Sherlock version 4.5 system. Summed feature 3 contained C_16:1_ω7c and/or C_16:1_ω6c and summed feature 8 contained C_18:1_ω7c. The database used was TSBA6.

### PHB biosynthetic pathway

**Figure 4:**
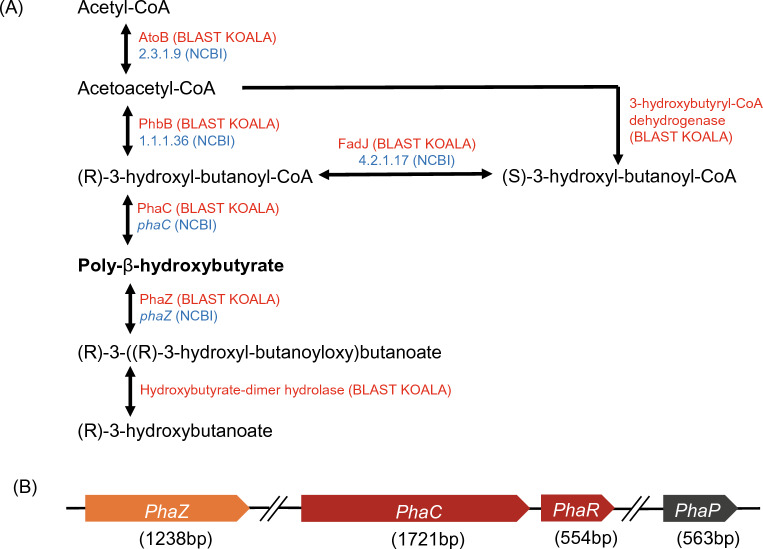
Polyhydroxybutyrate (PHB) biosynthetic pathway and polyhydroxyalkanoate (PHA) synthase gene cluster in DM-R-R2A-13^T^. (**A**) PHB biosynthetic pathway in strain DM-R-R2A-13^T^. AtoB, acetyl-CoA C-acetyltransferase; PhbB, Acetoacetyl-CoA reductase; PhaC, PHA synthase. Proteins identified in BlastKOALA analysis are marked in red with their names, and proteins identified in NCBI analysis are marked in blue with ID numbers. (**B**) PHA synthase gene cluster in strain DM-R-R2A-13^T^. The *phaC*, class I poly(R)-hydroxyalkanoic acid synthase; *phaR*, PHA synthesis repressor; *phaP*, TIGR01841 family phasin (small protein associated with inclusions such as PHA granules); *phaZ*; PHA depolymerase.

Since the TEM micrographs showed bacterial cells containing PHB granules in their cytoplasm, we performed a comprehensive analysis, including BlastKOALA analysis and NCBI PGAP analysis on the complete genome. The homopolymer PHB is one of the most extensively investigated compounds among the polyhydroxyalkanoates (PHA) family^41^. The BlastKOALA analysis indicated that the butanoate metabolism pathway is associated with the PHB biosynthesis pathway in DM-R-R2A-13^T^. The PHB biosynthesis process begins with the condensation of two acetyl-CoA units to form acetoacetyl-CoA by acetyl-CoA C-acetyltransferase (AtoB). Subsequently, PhaB reduces it to 3-hydroxyl-butanoyl-CoA. This resultant compound serves as a monomer that is polymerized into PHB by either PhaC or PhaE, depending on the organism^30^ (Fig. 4A).

### PHA-associated gene cluster

The enzyme responsible for PHB synthesis is PhaC, which polymerizes monomers into PHA polymer. PhaC’s substrate specificity determines the type of monomers that are incorporated into the PHA chain. There are four classes of PHA synthase, each with unique characteristics^31^. Class I and II PHA synthases are composed solely of PhaC. Class I PHA synthases, such as those found in *Cupriavidus necator* (formerly *Ralstonia eutropha*), exhibit a preference for CoA thioesters of (R)-3-hydroxy fatty acids that have 3–5 carbon atoms. On the other hand, class II PHA synthases, as seen in *Pseudomonas aeruginosa*, tend to favor CoA thioesters of (R)-3-hydroxy fatty acids that have 6–14 carbon atoms^32–34^. Class III PHA synthases, found in *Allochromatium vinosum*, are composed of two distinct enzymes, PhaC and PhaE, and they prefer CoA thioesters of (R)-3-hydroxy fatty acids with 3–5 carbon atoms^35,36^. In class IV PHA synthases, such as those found in *Bacillus megaterium*, there are two distinct enzymes, PhaC and PhaR, where PhaR substitutes PhaE in class III PHA synthases^37,38^. NCBI-based genome annotation of strain DM-R-R2A-13^T^ identified multiple genes associated with PHA metabolism, including *phaC*, *phaR*, *phaZ*, and *phaP*. It is clear that the *phaC* gene in DM-R-R2A-13^T^ belongs to Class I (Fig. 4B). We utilized Cluster Omega multiple sequence alignment to compare the amino acid sequence similarity of PhaC and PhaR of strains representing each class (Fig. S5). The results indicated high similarity to PhaC of *C. necator,* which belongs to class 1 (Table S1).

### Polymer analysis

**Figure 5:**
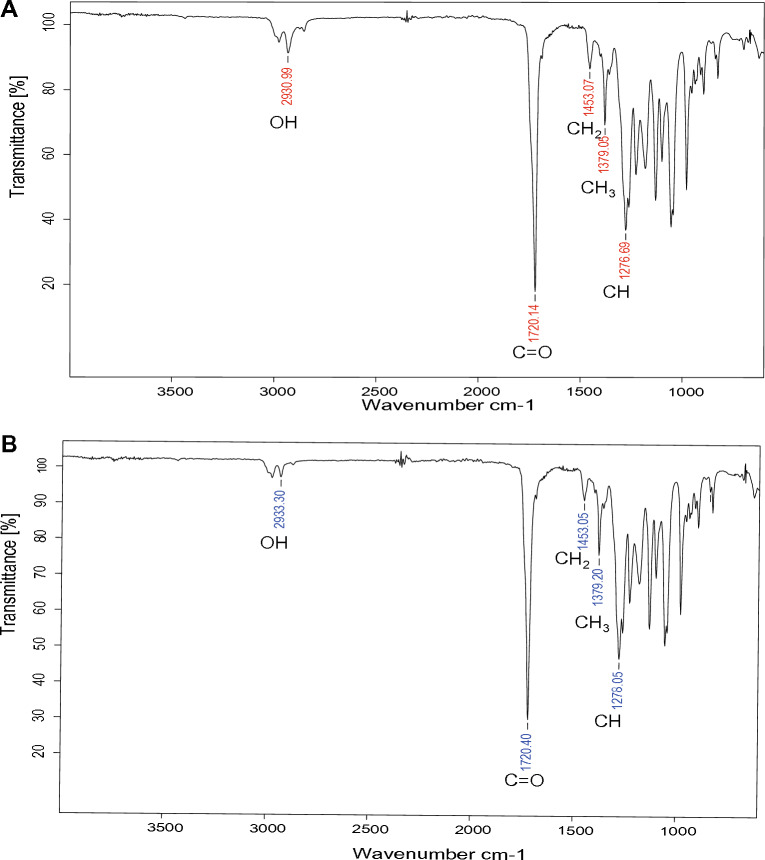
FT-IR spectra of PHB extracted from DM-R-R2A-13^T^. (**A**) FT-IR spectrum of PHB extracted from DM-R-R2A-13^T^ showing absorption peaks at 2930.99, 1720.14, 1453.07, 1379.05, and 1276.69 cm^−1^, corresponding to the OH, C=O, CH_2_, CH_3,_ and CH groups, respectively. (**B**) FT-IR spectrum of commercial PHB (Sigma-Aldrich).

FT-IR spectrum of PHB extracted from DM-R-R2A-13^T^ exhibited several absorption peaks. These include an OH group peak at 2930 cm^−1^, C=O stretching of an ester group peaks at 1720 cm^−1^, aliphatic –CH_2_ and –CH_3_ group peaks at 1453 cm^−1^ and 1379 cm^−1^, respectively, and –CH group peaks at 1276 cm^−1^ (Fig. 5A). The absorption peaks of the extracted PHB were similar to those of commercial PHB, as shown in Fig. 5B.

A ^1^H NMR analysis was utilized to analyze the chemical structure of PHB synthesized by the strain DM-R-R2A-13^T^. The ^1^H NMR spectrum had peaks at 1.26–1.28 ppm (-CH3), 2.45–2.62 ppm (–CH_2_), and 5.24–5.28 ppm (–CH) (Fig. S6A), confirming the chemical structure of PHB^39^. Additionally, the ^13^C NMR spectrum was analyzed to confirm the extracted PHB’s structure (Fig. S6B). The peaks corresponded to various carbon atoms within the PHB structure, including peaks at 169.15 ppm (C=O), 67.61 ppm (–CH), 40.79 ppm (–CH2), and 19.76 ppm (–CH3).

### Effect of carbon source on PHB production

**Figure 6:**
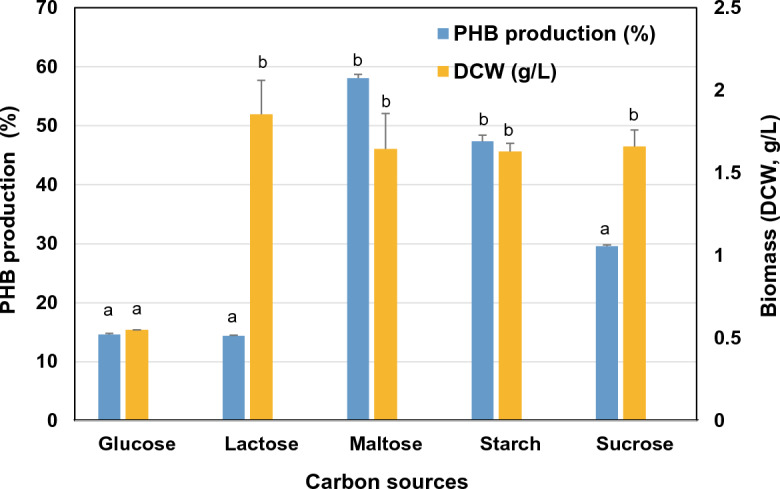
Effect of different carbon sources on PHB production and biomass (DCW) of DM-R-R2A-13^T^. Comparison of PHB production (%) and biomass (DCW, g/L) in MM medium with 2% (w/v) of different carbon sources. Error bars indicate standard error (n = 3). Means followed by different letters are significantly different at *P* < 0.05 according to one-way ANOVA.

The study examined whether the PHB production of DM-R-R2A-13^T^ was influenced by different carbon sources such as glucose, maltose, lactose, sucrose, fructose and starch. DM-R-R2A-13^T^ was cultured for 96 h in mineral salts medium supplemented with various 2% of carbon sources (glucose, maltose, lactose, sucrose, and starch), whereas bacterial growth was inhibited in the presence of 2% fructose. Results indicated that maltose (58.06% per DCW) resulted in the highest PHB content, followed by starch (47.37% per DCW) and sucrose (29.57% per DCW). However, the PHB content of 14.62% and 14.38% were obtained from glucose and lactose, respectively (Fig. 6). Previous studies have reported the production of PHB using starch (*M. albidiflava*, *M. aurea*, *M. brevitalea*, *M. aerilata*, *M. lutea*, *M. dura*, and *M. plicata*^40^) or glycerol (*M. plicata*^41^) as the carbon source, but strain DM-R-R2A-13^T^ utilized maltose as the major carbon source.

## Conclusion

In this study, we investigated strain DM-R-R2A-13^T^, which was obtained from the plant *Cannabis sativa* L. ‘Cheungsam’. Based on a polyphasic taxonomic approach that integrates phenotypic, chemotaxonomic, and phylogenomic characteristics, this strain exhibiting distinct phylogenetic lineages has been designated as novel species. Consequently, it has been assigned the name *Massilia endophyticus* sp. nov. We observed that the bacterium contained granules of PHA, indicating its potential for bioplastic production. The analysis of the genome revealed the presence of PHA synthase genes (*phaC*, *phaR*, *phaP*, and *phaZ*), further supporting its capability for biopolymer synthesis. Through NMR and FT-IR analyses, the biopolymer was identified as poly-3-hydroxylbutyrate (PHB). Notably, when maltose was utilized as the carbon source, the strain exhibited the highest PHB production rate of up to 58.06% of its dry cell weight.

### Description of *Massilia endophytica* sp. nov.

*Massilia endophyticus* (en.do.phy’ti.ca. Gr. pref. *endo-*, within; Gr. neut. n. *phyton* plant; L. fem. suff. *-ica*, adjectival suffix used with the sense of belonging to; N.L. fem. adj. *endophytica* within plants; *endophytic* pertaining to the original isolation from plant tissues).

Cells are Gram-negative, rod-shaped, aerobic, and motile. The colonies on R2A agar plates are circular, convex, and light pink in color, with a size of 1.5–2.0 µm after 2 days of incubation at 30 °C. The strain grows optimally at temperatures of 30–37 °C (it can grow at 15–40 °C) and pH values ranging from 6.0 to 7.5 (it can grow at pH 5.5–9.0), with optimal growth in the absence of NaCl. The strain showed sensitivity to kanamycin, tetracycline, and nalidixic acid in the antibiotic sensitivity tests. Catalase and oxidase are positive. Positive results were observed for alkaline phosphatase, esterase (C4), esterase lipase (C8), leucine arylamidase, valine arylamidase, cystine arylamidase, acid phosphatase, naphthol-AS-BI-phosphohydrolase, and β-glucosidase. Lipase (C14), trypsin, and α-chymotrypsin showed weak activity. Conversely, α-galactosidase, β-galactosidase, β-glucuronidase, α-glucosidase, *N*-acetyl-β-glucosaminidase, α-mannosidase, and α-fucosidase were negative according to the API ZYM strips. The API 20 NE results showed positive assimilation for potassium nitrate, esculin ferric citrate, L-arabinose, D-mannose, *N*-acetyl-glucosamine, and D-maltose. However, the assimilation of malic acid was weak. The DNA G + C content of the type strain is 64.6%. The dominant fatty acids are C_16:0_ and summed feature 3 (C_16:1_ω7c and/or C_16:1_ω6c). The major polar lipids are phosphatidylethanolamine (PE), phosphatidylglycerol (PG), and diphosphatidylglycerol (DPG). The predominant ubiquinone is Q-8.

The type strain DM-R-R2A-13^T^ (= KCTC 92,072^T^, GDMCC 1.2920^T^) was isolated from *Cannabis sativa*. L from Andong, Republic of Korea. The GenBank accession numbers for the 16S rRNA and the whole genome sequence of strain DM-R-R2A-13^T^ are OL314544 and CP088952, respectively.

## Materials and methods

### Sample collection and bacterial isolation

The strain DM-R-R2A-13^T^ was obtained from a sample of *Cannabis sativa* in Andong, Republic of Korea. To isolate the strain, leaf samples (5.3 g) and root samples (6.3 g) were subjected to surface-sterilization using 1% sodium hypochlorite for 20 min followed by 70% ethanol for 10 s, and then rinsed with sterile water. The sterilized samples were ground with 25 mL distilled water and spread onto plates containing Reasoner’s 2A (R2A) medium after serial dilution. The plates were incubated at 25℃ for 3 days, after which single colonies were isolated by transferring them onto new plates containing R2A medium and re-incubating. The strains were deposited at two culture collections: the Korean Collection for Type Cultures (KCTC) and the Guangdong Microbial Culture Collection Center (GDMCC), with accession numbers KCTC 92072^T^ and GDMCC 1.2920^T^, respectively.

### Phylogenetic analysis

To amplify the 16S rRNA gene of strain DM-R-R2A-13^T^, we used universal primers 518F and 800R as previously described^42^. The species with the closest match to the DM-R-R2A-13^T^ 16S rRNA gene sequence was obtained from the EzBioCloud server (http://www.ezbiocloud.net)^43^. Multiple sequence alignment was performed using all valid published members of the *Oxalobacteraceae* family with BioEdit (v.7.2.5). The nearly complete 16S rRNA gene sequence was submitted to the GenBank database (GenBank accession number, OL314544). To generate a phylogenetic tree, we employed the neighbor-joining (NJ), minimum evolution (ME), and maximum likelihood (ML) methods in MEGA X with 1000 bootstrap replications^44^. *Oxalobacter formigenes* U49757.2^T^ was used as the outgroup.

### Genomic sequencing and annotation

Genomic DNA was extracted for whole-genome sequencing using the protocol developed by Wilson et al.^45^. The sequencing was carried out at Macrogen, Inc. (Daejeon, Republic of Korea) using a PacBio Sequel/Sequel 2 system and an Illumina platform. The resulting data were assembled using a SMRT Link (v.8) de novo assembler. To determine the digital DNA–DNA hybridization (dDDH) and average nucleotide identity (ANI) values between DM-R-R2A-13^T^ and closely related strains, we employed the Genome-to-Genome Distance Calculation (GGDC) web server (http://ggdc.dsmz.de/) and EzBioCloud ANI calculator (www.ezbiocloud.net/tools/ani)^46,47^,utilizing the BLAST method. Metabolic features and pathways were determined by utilizing BlastKOALA, a tool that utilizes the Kyoto Encyclopedia of Genes and Genomes (KEGG) orthology system^48^. Genome annotation was also performed using the National Center for Biotechnology Information (NCBI) Prokaryotic Genome Annotation Pipeline^49^. A whole-genome phylogenetic tree was created using the up-to-date bacterial core gene set and pipeline (UBCG) as described by Na et al.^50^. *Oxalobacter formigens* U49757.2^T^ was used as the outgroup.

### Phenotypic and chemotaxonomic traits

Strain DM-R-R2A-13^T^ was cultivated on various growth media, including Luria–Bertani agar (LBA, Difco), marine agar 2216 (MA, Difco), malt extract agar (MEA, Difco), potato dextrose agar (PDA, Difco), Reasoner’s 2A agar (R2A, MB Cell), and tryptic soy agar (TSA, Difco). Growth in R2A broth was measured at various temperatures (4, 10, 15, 20, 25, 30, 35, 37, 40, 45, 50, and 55 °C), pH values (pH 3.0–12.0, at intervals of 0.5 pH unit), and NaCl concentrations (0, 0.05, 0.1, 0.15, 0.2, 0.5, and 1.0%; w/v) after 2 days of incubation. Gram staining was conducted using a Gram Stain Solution kit (Difco). Catalase activity was tested using 1.0% tetramethyl-*p*-phenylendiamine (Oxidase Reagent Kit, bioMérieux). Anaerobic growth was determined by incubating the strain on R2A medium in an anaerobic chamber with an 86% N_2_, 7% CO_2,_ and 7% H_2_ atmosphere at 30 °C for 14 days.

Cell motility was evaluated using R2A medium containing 0.4% agar, and the presence of flagella was assessed by scanning electron microscopy (SEM); the cells were fixed with 2% glutaraldehyde and 2% paraformaldehyde in 50 mM cacodylate buffer (pH 7.4) for 1 h at 4 °C. Antibiotic sensitivity was determined using antibiotic discs (BD BBL™) containing one of the following antibiotics: (μg/disc): chloramphenicol (30), kanamycin (30), nalidixic acid (30), nitrofurantoin (300), tetracycline (30), and penicillin (10 U) on R2A medium. Enzyme activity and assimilation activity were determined using API 20 NE (bioMérieux) and API ZYM (bioMérieux), respectively.

To determine the fatty acid content, strain DM-R-R2A-13^T^ and reference strains (KCTC 23585^T^, DSM 104676^T^, and DSM 16850^T^) were cultivated for 2 days on R2A. Cells harvested from the culture were subjected to fatty acid methyl ester extraction following the standard MIDI instructions (Sherlock Microbial Identification System v.6.0), and the resulting samples were analyzed using gas chromatography (model 6890N; Agilent) with the Microbial Identification software package (TSBA database v.6.0)^51^. Freeze-dried cells (100 mg) were employed to extract isoprenoid quinones using a methanol/isopropyl ether (3:1, v/v) mixture, followed by thin-layer chromatography analysis, as previously described by Colins et al.^52^. Polar lipids were extracted from freeze-dried cells (100 mg) using a chloroform/methanol mixture (2:1, v/v). The separation of polar lipids was achieved by two-dimensional thin-layer chromatography (silica gel, 20 × 20 cm; Merck) followed by spraying with phosphomolybdic acid to identify polar lipids^53^.

### Transmission electron microscopy (TEM)

The samples were fixed with a solution containing 2% glutaraldehyde and 2% paraformaldehyde in 50 mM cacodylate buffer (pH 7.4) for 1 h at 4 °C, followed by post-fixation with 2% osmium tetroxide and 3% potassium hexacyanoferrate for 40 min. Subsequently, the samples were dehydrated in a graded series of ethanol, with each dilution lasting for 10 min, the samples were immersed in a mixture of 100% ethanol and LR white resin (in ratios of 2:1, 1:1, or 1:2) for 10 min, and then transferred to pure LR white resin for an additional 15 min. The samples were transferred to a dry capsule or mold, which was filled with embedding resin. The resin was cured in a 60 °C oven for 24 h. The samples were then subjected to ultra-thin sectioning (80 nm), placed on a copper grid, stained with uranyl acetate and lead citrate, and viewed using TEM (JEM-2100F, JEOL) at an accelerating voltage of 200 kV at the Korea Basic Science Institute (Chuncheon, Republic of Korea)^54^.

### Extraction and purification of PHB

The extraction and purification of PHB followed the experimental method detailed by Trakunjae et al.^55^. For the extraction of PHB, freeze-dried cells (1 g) were dissolved in 100 mL of chloroform and allowed to stand at room temperature for 4 days. Afterward, the solutions were filtered using ADVANTEC filter paper (90 mm) to eliminate any cellular debris and obtain a purified solution. Subsequently, the filtrate was slowly added dropwise to 100 mL of cold-methanol (Honeywell, USA). The resulting purified polymer was air-dried for 3 days and collected for Fourier transform infrared (FT-IR) spectroscopy and nuclear magnetic resonance (NMR) analysis to confirm its chemical structure as polyhydroxylbutyrate (PHB).

### FT-IR spectroscopy

FT-IR spectroscopy was conducted using a Bruker Lumos II FT-IR microscope at Koptri Inc. (Seoul, Republic of Korea). The samples were scanned within a wavenumber range of 4000–400 cm^−1^ at a resolution of 4 cm^−1^, with a total of 32 scans per sample.

### NMR analysis

Both ^1^H NMR and ^13^C NMR spectra were obtained using a Bruker Avance III™ HD 600 MHz at Koptri Inc. (Seoul, Republic of Korea). 0.1 g of bacterial-isolated PHB samples, as well as the standard PHB (Sigma-Aldrich, USA), were dissolved in chloroform. Tetramethylsilane (TMS) signals were used as reference standards for the chemical shifts observed in the ^1^H and ^13^C spectra.

### Effect of carbon source on PHB production

PHB production by strain DM-R-R2A-13^T^ was explored in a 250-mL Erlenmeyer flask containing 100 mL of mineral salt medium (MM medium). This medium consisted of Na_2_HPO_4_·12H_2_O, 9 g/L; KH_2_PO_4_, 1.5 g/L; NH_4_Cl, 1 g, MgSO_4_·7H_2_O, 0.2 g/L; CaCl_2_, 0.02 g/L; ferric ammonium citrate, 0.0012 g/L, and 100 μL/L of trace element (TE) solutions. The TE solution comprised: ZnSO_4_, 1 g/L; MnCl_2_·4H_2_O, 0.3 g/L; H_3_BO_3_, 3 g/L; CoCl_2_·2H_2_, 0.1 g/L; NiCl_2_·6H_2_, 0.2 g/L; NaMoO_4_·2H_2_, 0.3 g/L. Furthermore, 2% of various carbon sources (glucose, starch, maltose, sucrose, fructose or lactose) were added to the medium. The flasks were incubated in a shaker at 150 rpm and 30 °C for 96 h. Cells were obtained after centrifuging the culture at 8,000 rpm for 15 min, and the samples were freeze-dried for at least 24 h. Lyophilization was deemed complete when the cell pellets were dry. All experiments were performed in triplicate.

### Gas chromatography (GC)

To quantify the PHB, GC was used with a DB-23 capillary column (60 m × 250 μm × 0.25 μm). The PHB was converted to 3-hydroxybutyryl methyl ester (3-HBME), a stable monomer, in the GC column using the acidic methanolysis method^56^. Freeze-dried cells (10 mg) were mixed with 1 mL chloroform and 1 mL methanol containing 15% (v/v) H_2_SO_4_ and incubated for 2.5 h at 100 °C. After cooling the samples on ice for 5 min, 1 mL deionized water and 1 mL chloroform containing 0.2% (v/v) methyl benzoate as an internal standard were added. The mixture was vortexed and the organic (bottom) phase containing methylesters was used for PHB analysis. Commercial PHB (Sigma-Aldrich) was used as the reference standard. The GC configuration parameters were set as follows: injection volume, 1 μL; split ratio, 1:25; injection temperature, 250 °C; column oven temperature, 80 °C for 2 min; and temperature ramp, 10 °C/min^−1^ and 245 °C for 1 min. The detection was performed using a flame ionization detector at 275 °C.

### Quantification of PHB

The weight of PHB was estimated using a standard curve constructed based on commercial PHB (Sigma-Aldrich) at various concentrations (2, 4, 6, and 8 mg) following the formula used by Jannina et al.^57^. The normalized area of 3-HBME peaks was calculated using the following formula:

$$Normalized \, \;area = \frac{Area\; of\; 3HBME*\;Area\; of\; external \;standard}{{Area \;of \;internal \;standard}}$$

The weight of PHB in a sample with various carbon sources was calculated using the following formula:

$${ }Weight\; \, of\; \, PHB\; \, in\; \, sample = \frac{Normalized \;area \;of\; sample\; - b}{a}$$

a is a standard curve’s slope and b is a standard curve’s y-intercept.

The percentage of PHB per dry cell weight (DCW) was calculated using the following formula:

$$PHB\; \, content \, \left( {\% \, per \, dry \,cell \, weight} \right) = Weight\; \, of\; \, PHB \, \;in\; \, sample*\frac{100}{{weighted \;cells}}$$

### Statistical analysis

The data were analyzed using one-way analysis of variance (ANOVA) in GraphPad Prism v.9, and the results are presented as mean ± standard deviation. Error bars indicate standard error (n = 3). A significant level of *p*-value ≤ 0.05 was used to determine statistical significance.

### Ethics approval

Experimental research and field studies on plants, including the collection of plant material, complying with relevant institutional, national, and international guidelines and legislation.

### Supplementary Information

Supplementary Information.

## Data Availability

Strain DM-R-R2A-13^T^ can be obtained from two culture collections, the Korean Collection for Type Cultures (KCTC 92072^T^) and the Guangdong Microbial Culture Collection Center (GDMCC 1.2920^T^). The 16S rRNA gene sequence of strain DM-R-R2A-13^T^ is available under GenBank accession number OL314544, while the whole-genome sequence is CP088952. The associated BioSample and BioProject accession numbers are SAMN23170019 and PRJNA678113, respectively. The taxonomy ID for strain DM-R-R2A-13^T^ is 2,899,220.
