# Supplementary material for: Bioplastic (poly-3-hydroxybutyrate)-producing Massilia endophytica sp. nov., isolated from Cannabis sativa L. ‘Cheungsam’
Source: Sci Rep. 2023 Oct 18;13:17767. doi: 10.1038/s41598-023-44976-w (PMC10584911; doi:10.1038/s41598-023-44976-w)
Supplement: Supplementary file 1 — Supplementary Information. [file 41598_2023_44976_MOESM1_ESM.pdf]

Supplementary material

Bioplastic (poly-3-hydroxybutyrate) producing *Massilia endophytica* sp. nov., isolated from *Cannabis sativa* L. 'Cheungsam'

Doeun Jeon[1,2], Lingmin Jiang[1], Ki-Hyun Kim[1], Yuxin Peng[1], Donghyun Cho[1], Rae-Dong Jeong[2], Cha Young Kim[1], Jae Cheol Jeong[1], and Jiyoung Lee[1,3*]

[1]Korean Collection for Type Cultures (KCTC), Biological Resource Center, Korea Research Institute of Bioscience and Biotechnology, Jeongeup 56212, Republic of Korea

[2]Department of Applied Biology, Chonnam National University, Gwangju 61186, Republic of Korea

[3] Department of Biosystems and Bioengineering, KRIBB School of Biotechnology, University of Science and Technology (UST), Yuseong, Daejeon 34113, Republic of Korea

* **Correspondence: Jiyoung Lee**, jiyoung1@kribb.re.kr

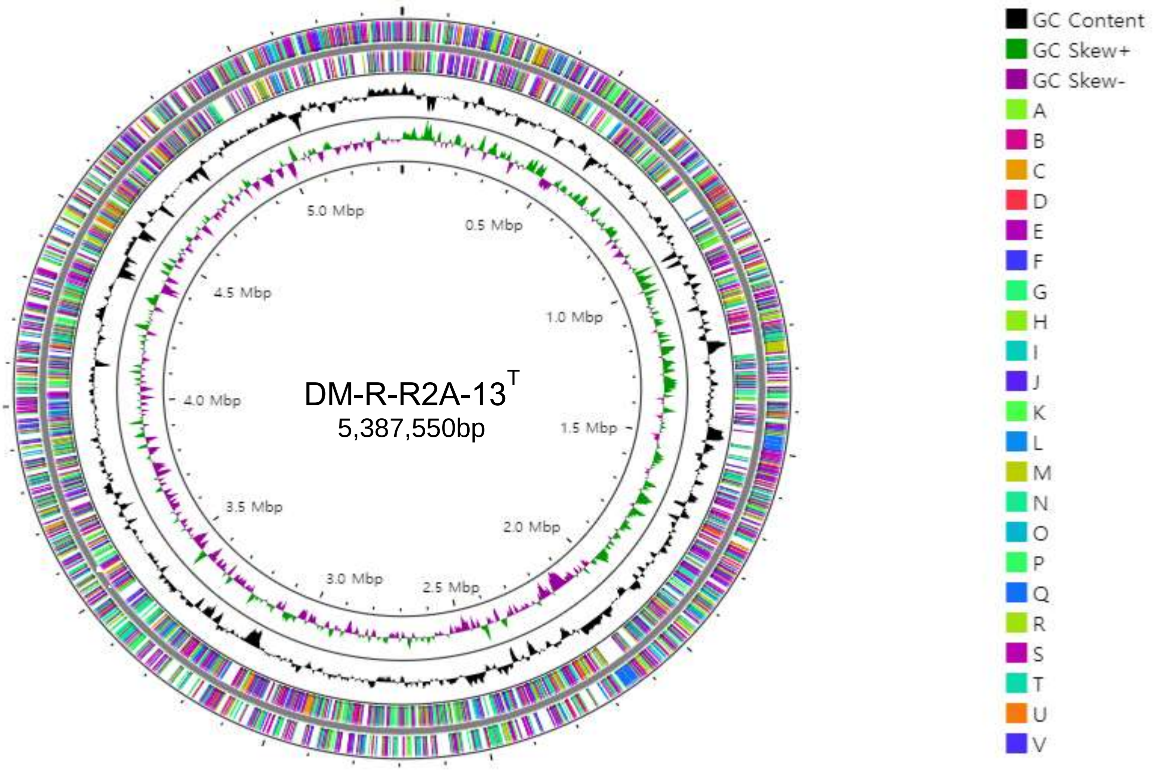

Fig. S1. Genome map of the DM-R-R2A-13^{T} generated using CGView. Distinctive features illustrated from out to inner rings. Ring 1 and ring 2 depict forward and reverse Cluster Orthologous Group (COG) annotation, respectively. Ring 3 illustrates the G+C % content plot, while ring 4 depicts the GC skew. The COG categories are: A, RNA processing and modification; B, chromatin structure and dynamics; C, energy production and conversion; D, cell cycle control, cell division, chromosome partitioning; E, amino acid transport and metabolism; F, nucleotide transport and metabolism; G, carbohydrate transport and metabolism; H, coenzyme transport and metabolism; I, lipid transport and metabolism; J, translation, ribosomal structure and biogenesis; K, transcription; L, replication, recombination and repair; M, cell wall/membrane/envelope biogenesis; N, cell motility; O, posttranslational modification, protein turnover, chaperones; P, inorganic ion transport and metabolism; Q, secondary metabolites biosynthesis, transport and catabolism; R, general function prediction only; S, function unknown; T, signal transduction mechanisms; U, intracellular trafficking, secretion, and vesicular transport; V, defense mechanisms.

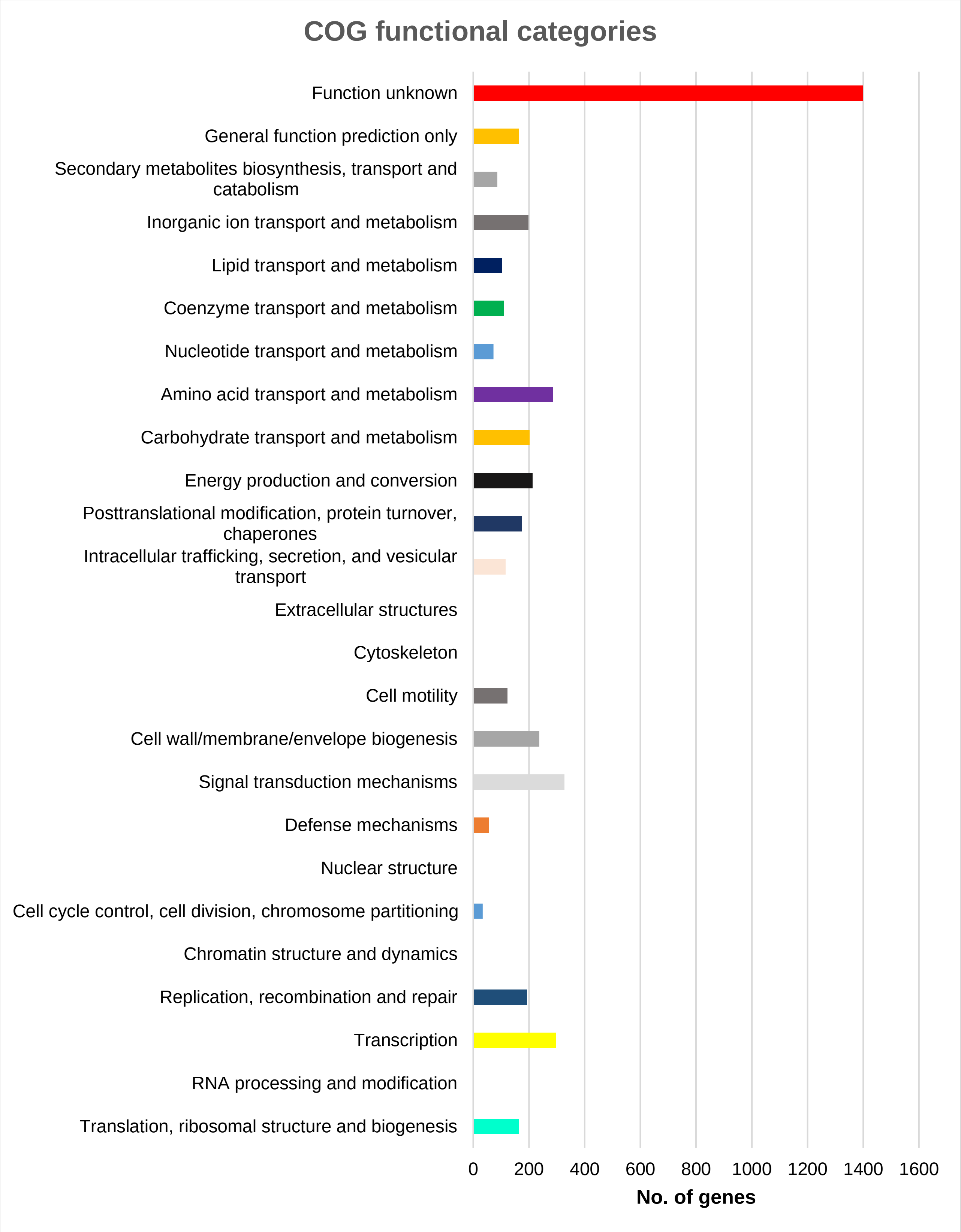

Fig. S2. COG classification of strain DM-R-R2A-13^{T} genome. Of the 4,569 transcripts identified using the eggNOG database, 3,170 were categorized into 25 COG clusters.

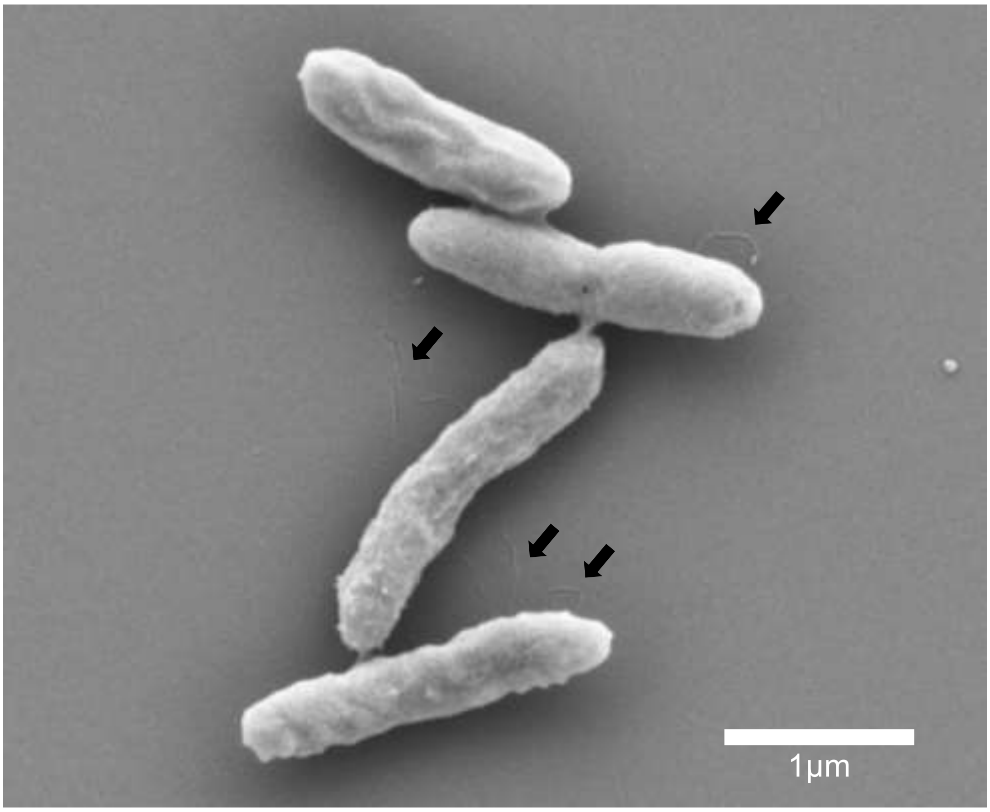

Fig. S3. Scanning electron microscopy (SEM) image of DM-R-R2A-13^T grown in R2A medium at 30°C. Scale bar, 1 μm.

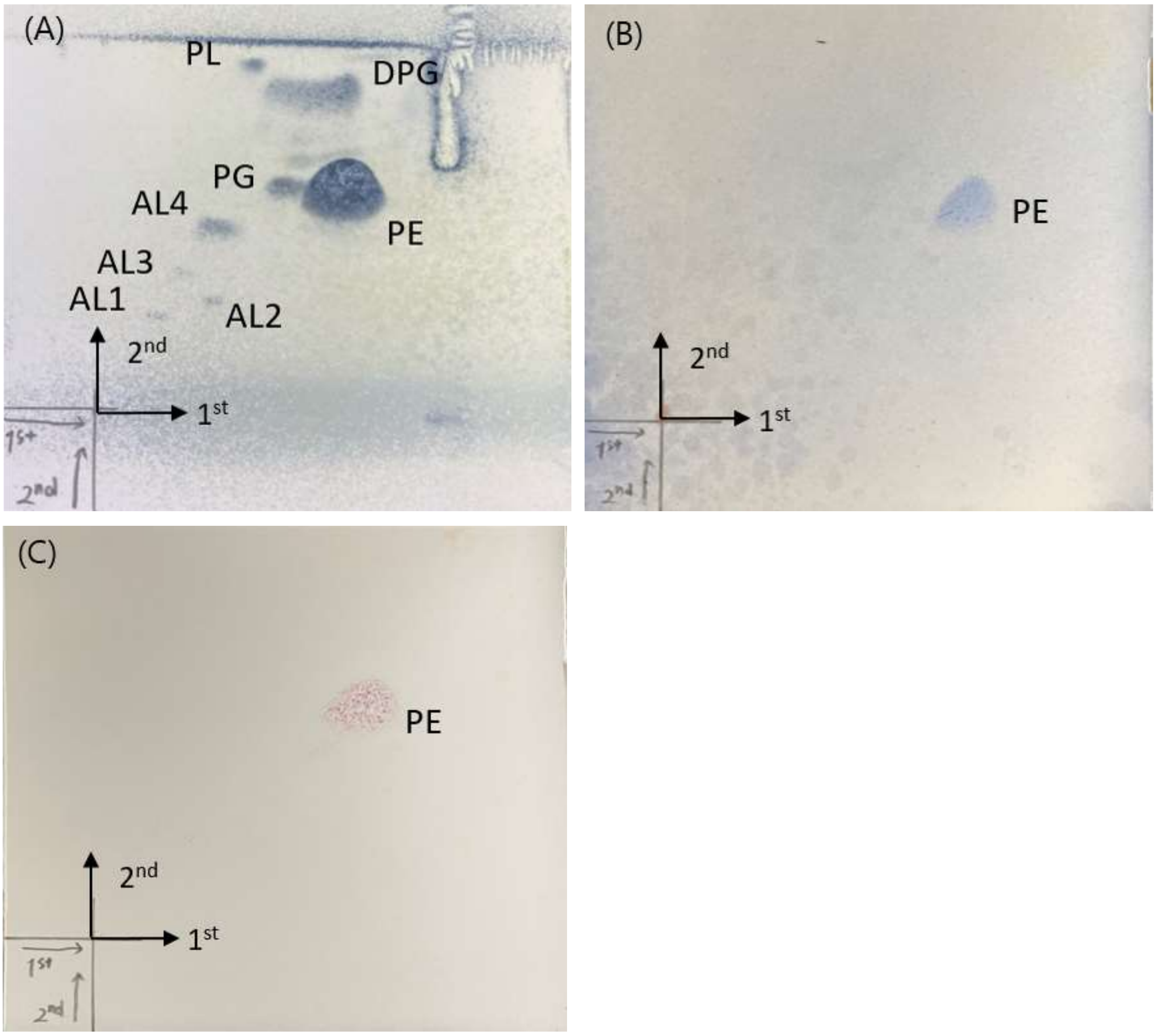

Fig. S4. Two-dimensional thin-layer chromatography of polar lipids of strain DM-R-R2A-13^{T}. (A) Total lipids were detected by spraying with phosphomolybdic acid. (B) Phospholipids (PL) were detected by spraying with molybdenum blue. (C) Aminolipids (AL) were detected by spraying with ninhydrin. Diphosphatidylglycerol (DPG), phosphatidylethanolamine (PE), phosphatidylglycerol (PG), one unidentified PL, and four unidentified AL were observed.

```
M. endophytica DM-R-R2A-13 LTKGLANMLADMQKGHISLSDEKAFEVGRNLATTEGQVVYENELFQLLQYKPLTEN--VK   219
R. necator H16             LRAGVRNMMEDLTRGKISQTDESAFEVGRNVAVTEGAVVFENEYFQLLQYKPLTDK--VH   236
B. megaterium              MPSEYKSSARRFKRAYEIMTAEAEP----EVGLTPKEVIWKKNKAKLYRYTPVKDN--LH   69
A. vinosum DSM180          LTQEMLDYSRKLGQGMENLLNAEAI----DTGVSPKQAVYSEDKLVLYRYDRPEGAPEAQ   66
                           :     .    : :.              : . :   .::.::   * :*         :

M. endophytica DM-R-R2A-13 QRPLLMVPPCINKYYILDLQPENSLVRYAVEQGNTVFLISWRNPNQTLAKTSWDDYVDDG   279
R. necator H16             ARPLLMVPPCINKYYILDLQPESSLVRHVVEQGHTVFLVSWRNPDASMAGSTWDDYIEHA   296
B. megaterium              KTPILLVYALINKPYILDLTPGNSLVEYLLNRGFDVYLLDWGTPGLEDSNMKLDDYIVDY   129
A. vinosum DSM180          PVPLLIVYALVNRPYMTDIQEDRSTIKGLLATGQDVYLIDWGYPDQADRALTLDDYINGY   126
                             *:*:*   :*: *: *:    * :.  :  *  *:*:.*  *.      . ***:

M. endophytica DM-R-R2A-13 VVNAINICKAITKEEKLNVLGFCVGGTLLSTALAVMAARGEQPAASVTLLTTFLDFSDTG   339
R. necator H16             AIRAIEVARDISGQDKINVLGFCVGGTIVSTALAVLAARGEHPAASVTLLTTLLDFADTG   356
B. megaterium              IPKAAKKVLRTSKSPDLSVLGYCMGGTMTSIFAA---LNEDLPIKNLIFMTSPFDFSDTG   186
A. vinosum DSM180          IDRCVDYLREAHGVDKVNLLGICQGGAFSLMYSA---LHPDK-VRNLVTMVTPVDFKTPD   182
                             .. .         .:.:** * **::     *    . :    .:  :.: .**   .

M. endophytica DM-R-R2A-13 -VLNVFVDEPQVMLREQTLAAGG-----LMPGRDLASTFSSLRPNDLVWNYVQSNYLKGN   393
R. necator H16             -ILDVFVDEGHVQLREATLGGGAGAPCALLRGLELANTFSFLRPNDLVWNYVVDNYLKGN   415
B. megaterium              -LYGAFLDDRYFNLDKAVDTFG------NIPPEMIDFGNKMLKPITNFYGPYVTLVDRSE   239
A. vinosum DSM180          NLLSAWVQ--NVDIDLAVDTMG------NIPGELLNWTFLSLKPFSLTGQKYVNMVDLLD   234
                            : ..:::   . :   .   *       :    :      *:*               :

M. endophytica DM-R-R2A-13 EP----PPFDLLYWNSDSTNLPGPMFCWYLRNTYLENKLKDPGRLKVAGEAVDLSKIDAP   449
R. necator H16             TP----VPFDLLFWNGDATNLPGPWYCWYLRHTYLQNELKVPGKLTVCGVPVDLASIDVP   471
B. megaterium              NQRFVESWKLMQKWVADGIPFAGEAYRQWIRDFYQQNKLI-NGELEVRGRKVDLKNIKAN   298
A. vinosum DSM180          DPDKVKNFLRMEKWIFDSPDQAGETFRQFIKDFYQNNGFL-NGGVVLGGQEVDLKDITCP   293
                                     :  *  *.    *  :  :::. * :* :   * : * *  *** .*
```

Fig. S5. Alignment of the PHA synthase (PhaC) from *M. endophytica* DM-R-R2A-13^{T} with those from the following strains: *R. eutropha* H16 (QCC00456.1), *B. megaterium megaterium* ATCC 14581 (QSF29105.1), and *A. vinosum* DSM 180 (ADC61033.1). The lipase box-like region is in red box. "*****" " means that the amino acids in that column are identical in all sequences in the alignment. "**:**" means that conserved substitutions have been observed. "**.**" means that semi-conserved substitutions are observed.

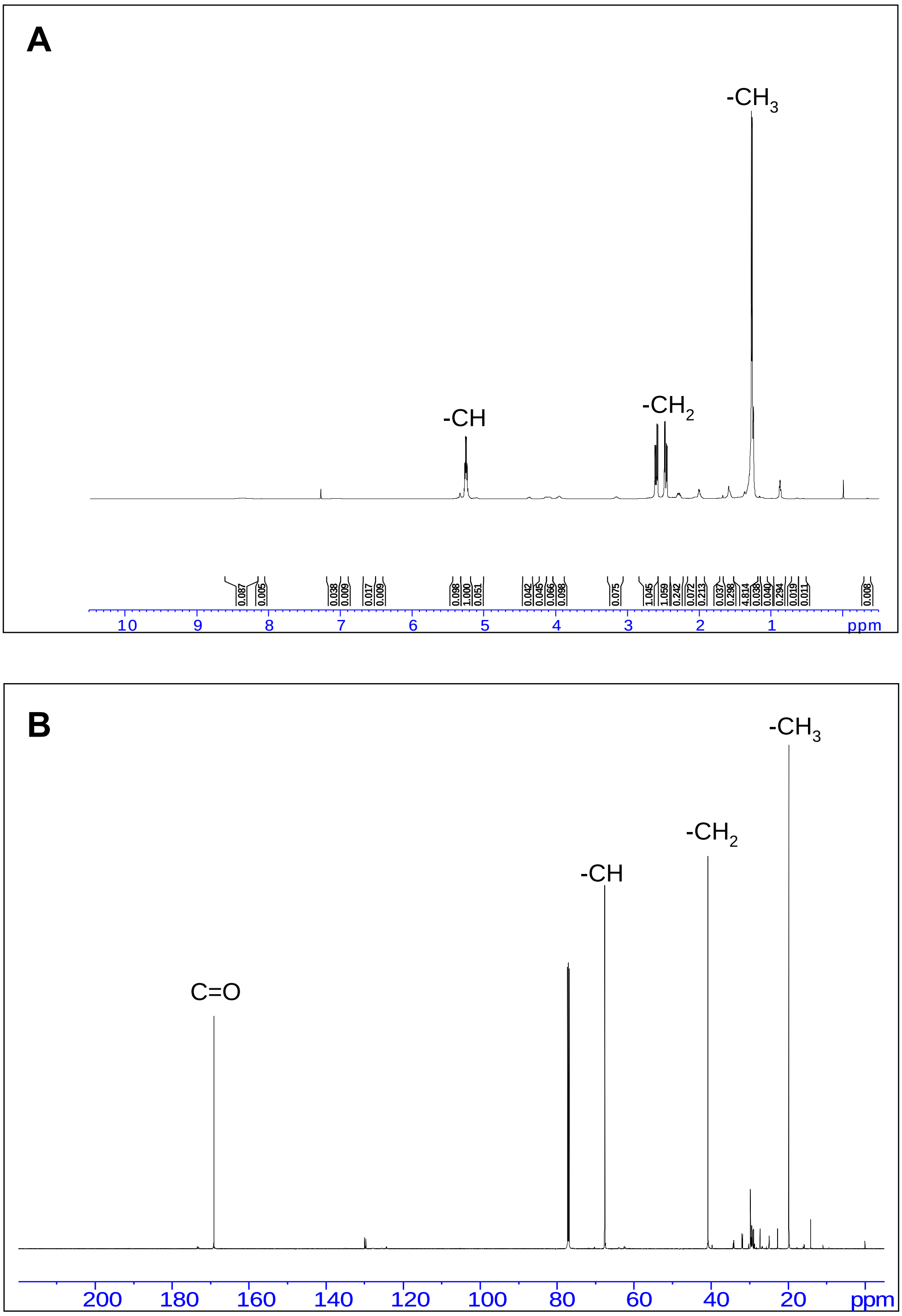

Fig. S6. NMR spectra of PHB extracted from DM-R-R2A-13^T (A) ^{1}H NMR spectrum has peaks at 1.26–1.28 ppm (-CH_3), 2.45–2.62 ppm (-CH_2), and 5.24–5.28 ppm (-CH). (B) ^{13}C NMR spectrum has peaks at 169.15, 67.61, 40.79, and 19.76 ppm, corresponding to the carbon atom of C=O, -CH, -CH_2, and -CH_3, respectively.

Table S1. Comparative analysis of PHA synthase in strain DM-R-R2A-13[T] and other bacterial proteins. The protein sequences were obtained from the National Center for Biotechnology Information (NCBI), and the percentage identity was calculated using Cluster Omega multiple sequence alignments (Clustal v.2.1).

Organism	PhaC		PhaR	
	GeneBank no.	Identity (%)	GeneBank no.	Identity (%)
R. eutropha H16[a]	QCC00456.1	60.88	QCC00459.1	72.53
B. megaterium ATCC 14581[b]	QSF29105.1	27.81	QSF29103.1	18.80
A. vinosum DSM 180[c]	ADC61033.1	25.00	ADC61036.1	44.90

[a] *R. eutropha* (=*Raltonia eutropha*, =*Cupriavidus necator*)

[b] *B. megaterium* (=*Bacillus megaterium*, =*Priestia megaterium*)

[c] A. vinosum (=*Allochromatium vinosum*)
